# Surgical vacuum filter-derived stromal cells are superior in proliferation to human bone marrow aspirate

**DOI:** 10.1186/s13287-019-1461-0

**Published:** 2019-11-21

**Authors:** Katharina Henze, Monika Herten, Marcel Haversath, André Busch, Sven Brandau, Alexander Hackel, Stefanie B. Flohé, Marcus Jäger

**Affiliations:** 1 Department of Orthopaedics and Trauma Surgery, University Hospital Essen, University of Duisburg-Essen, Hufelandstrasse 55, 45147 Essen, Germany; 2 Department of Otorhinolaryngology, University Hospital Essen, University of Duisburg-Essen, Hufelandstrasse 55, 45147 Essen, Germany; 3 Department of Orthopaedics, Trauma and Reconstructive Surgery, University of Duisburg Essen & St. Marien Hospital Mülheim an der Ruhr / Contilia, Kaiserstrasse 50, 45468 Mülheim/Ruhr, Germany

**Keywords:** Mesenchymal stromal cells, Bone regeneration, Cell saver, Filter, Bone marrow, Colony-forming units

## Abstract

**Background:**

During joint replacement, surgical vacuum suction guarantees a sufficient overview on the situs. We assume high concentrations of mesenchymal stromal cells (MSCs) on surgical vacuum filters.

We compared the in vitro proliferative and differentiation potency of cells from the following: (i) bone marrow (BM), (ii) cancellous bone (CB), (iii) vacuum filter (VF), and (iv) cell saver filtrate reservoir (SF) in 32 patients undergoing elective total hip replacement.

**Methods:**

Mononuclear cells (MNC) were isolated, and cell proliferation and colony-forming units (CFU) were measured. Adherent cells were characterized by flow cytometry for MSC surface markers. Cells were incubated with osteogenic, adipogenic, and chondrogenic stimuli. Cells were cytochemically stained and osteoblastic expression (RUNX-2, ALP, and BMP-2) investigated via qPCR.

**Results:**

Dependent on the source, initial MNC amount as well as CFU number was significantly different whereas generation time did not vary significantly. CFU numbers from VF were superior to those from SR, BM, and CB. The resulting amount of MSC from the respective source was highest in the vacuum filter followed by reservoir, aspirate, and cancellous bone. Cells from all groups could be differentiated into the three mesenchymal lines demonstrating their stemness nature. However, gene expression of osteoblastic markers did not differ significantly between the groups.

**Conclusion:**

We conclude that surgical vacuum filters are able to concentrate tissue with relevant amounts of MSCs. A new potent source of autologous regeneration material with clinical significance is identified. Further clinical studies have to elucidate the regenerative potential of this material in an autologous setting.

## Background

Besides regulation of the normal skeletal homeostasis including calcium-phosphate metabolism, hematopoiesis, and further immunological functions, the adult human bone marrow contains osteoprogenitor cells that play a crucial role in fracture healing and osteogenesis [1].

Clinical and experimental data suggest several different stimuli orchestrating the complex molecular interactions of bone healing. Representative players are the platelet-rich plasma (PRP) and its contained growth factors, different cytokines, and fibrin. Besides soluble agents, the carefully controlled balance between bone forming (osteoblast), bone resorbing (osteoclast), and other cell types is essential for physiological bone formation [2–7]. Bone fractures as well as osteotomies are associated with soft and hard tissue trauma. Especially in total hip replacement, relevant amounts of the bone marrow leave the acetabulum and femoral canal during preparation procedures. Moreover, surgical approaches are associated with relevant soft tissue trauma (ruptures of small vessels, elongation and strain of muscles, and damage of fat tissue). At least an undefined mixture of the bone marrow, small cancellous bone fragments, fat, hematoma, and additional soluble factors occurs at the bony implant site. In cementless total joint replacement, this “bone stew” gets in direct contact to the implant, binds to its surface, and determinates the preconditioning protein film (implant proteome) [8]. Together with inflammatory cytokines and nervous stimuli, this peri-implant microenvironment includes a strong osteogenic potential, associated with an increased risk for ectopic bone formation (non-hereditary heterotopic ossification, NHHO) [9–13]. However, relevant amount of this cytokine cocktail in the implant-side released tissue is removed by surgical suction.

In total joint replacement and other relevant orthopedic interventions, surgical vacuum suckers are frequently used. This technology allows not only a sufficient overview on the situs, but lowers as well the need for allogenic blood transfusions and its associated risks [14–18]. However, when applied intraoperatively, not only blood or hematoma is aspirated by the sucker but also other soluble or small tissue components within the operation site such as the “bone stew” are removed. Therefore, commercial vacuum suckers are combined with filters retaining tissue fragments and fibrin clots and discharge blood components for the downstream connected cell saver system [19]. Our hypothesis was that the filter contains relevant amounts of mesenchymal progenitor cells as well as the downstream cell saver reservoir. We investigated this question experimentally comparing the in vitro proliferative and differentiation potency of MSC from bone marrow aspirate (BM-MSC), cancellous bone-derived MSC (CB-MSC), vacuum filter-derived MSC (VF-MSC), and MSC from the cell saver filtrate reservoir (SR-MSCs) in 32 patients undergoing elective total hip replacement.

## Materials and methods

### Patients

Following a prospective design, the patient cohort consists of 32 patients (22 females, 10 males, mean age of 67.4 ± 10.1 years) with advanced osteoarthritis qualified for elective total hip replacement. Exclusion criteria were malignant or infectious diseases and an age under 18 years. Table 1 summarizes the patients’ characteristics. All patients were operated in supine position using a Bauer-Harding approach.

**Table 1:** Patients’ characteristics and comorbidities

Parameter	Number (%)
Patients	32 (100%)
Age (years), mean ± SD	67 ± 10.06
Gender female	22 (69.7%)
Comorbidities	
Cardiovascular disease	23 (71.9%)
Rheumatoid diseases	6 (18.8%)
Venous diseases	6 (18.8%)
Thyroid disorders	5 (15.6%)
Diabetes mellitus	3 (9.4%)
Renal insufficiency	3 (9.4%)
Osteoporosis	3 (9.4%)
Chronic obstructive pulmonary disease (COPD)	2 (6.3%)
Time of surgery (min), median [range]	82 [55–140]

### Cell harvesting methods

**Fig. 1:**
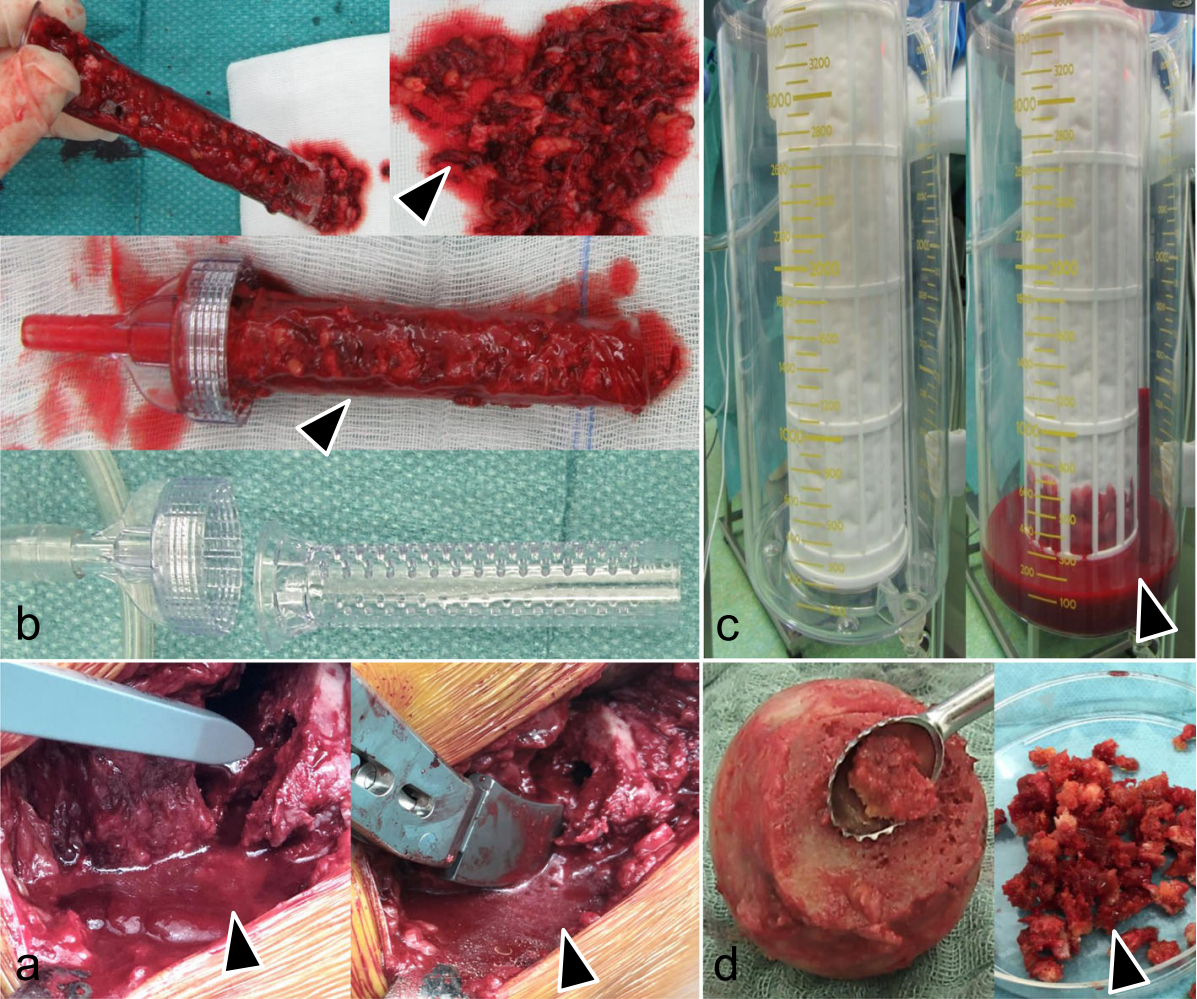
Tissue harvest during surgery. **a** Mixture of different liquid tissue components (arrow) released during implantation of a cementless titanium hip stem before aspiration by the surgical vacuum sucker. **b** Surgical vacuum filter handle: empty and with a vacuum filter clot (VF) (arrow). **c** Cell saver filtrate reservoir (SR): empty and with collected liquid (arrow). **d** Femur head before the removal of cancellous bone (CB) (arrow)

During surgery, four tissue samples were harvested and prepared for further in vitro cultivation (Fig. 1):

1. *B*one *m*arrow (BM): After femoral neck resection, the femur canal was accessible and a G 20 needle with a 10-mL syringe (Sterican® G20 × 1 1/2″ = 0.90 × 40 mm, B. Braun Melsungen, Germany) was inserted into the native femoral head before any probe or rasp were inserted. A volume of 3.3 ± 2 mL of the bone marrow was harvested by vacuum suction with the syringe and diluted with PBS up to a volume of 20 mL for Ficoll density gradient centrifugation.
2. *C*ancellous *b*one (CB) was removed from the femoral head by a sharp spoon: the spongy material was removed, weighed, and minced by surgical scissors and treated by collagenase D (final 1 mU/mL, Roche/Sigma-Aldrich, Deisenhofen, Germany) for 15 min at 37 °C and 6% CO_2_. Afterwards, solid tissue fragments were removed by rinsing with PBS using a cell strainer (nylon mesh with pore size 70 μm, Becton Dickinson, BD Bioscience, Heidelberg, Germany), followed by centrifugation with 600*g* for 15 min at RT. The cells were resuspended in 20 mL PBS for Ficoll density gradient centrifugation.
3. Surgical *v*acuum *f*ilter clot (VF): The fibrin clot adhered to the surface of the surgical vacuum filter was removed, and 2–3 g of the VF material was incubated with streptokinase (10,000 U per 5 g, Sigma-Aldrich, Deisenhofen, Germany) diluted 1:1 with PBS and mixed by a magnetic stirrer with 100 rpm for 15 min at RT for thrombolysis. The mixture was centrifuged at 600*g* for 10 min at RT, and the pellet was suspended in 20 mL PBS for density gradient centrifugation.
4. Cell *s*aver *r*eservoir (SR) (Dideco [Sorin] Electa Autotransfusion System Cell Saver, Dideco, Milan, Italy): 2–3 mL of the SR material (filtrate) was treated as described for VF (streptokinase treatment and density gradient, see above).

### Isolation of mesenchymal stromal cells

The isolation of MSC followed a standardized protocol based on Ficoll (Ficoll Paque™ Plus, density 1.078 g/mL, GE Healthcare, Freiburg, Germany) density gradient centrifugation as reported previously [20]. 10 ×  10^6^ mononucleated cells (MNC) of each probe were cultivated in T75 tissue flasks in low-glucose DMEM (Gibco, Life Technologies, Darmstadt, Germany) culture media containing 10% (v/v) fetal calf serum (FCS; Biochrome, Berlin, Germany), 100 U/mL penicillin, 0.1 mg/mL streptomycin, 2 mM-glutamax, and 1 mM sodium pyruvate (all from Sigma-Aldrich).

Following the International Society for Cellular Therapy’s (ISCT) minimal criteria to define mesenchymal stromal cells (MSCs), we choose plastic adherence, as well as appropriate surface marker expression and trilineage differentiation for MSC characterization [21–25].

### Generation time

The adherent cells were split and seeded in a density of 6.5 ± 2.5 × 10^3^ cells per cm^2^ in flasks (passage 1). After reaching a confluency of about 90%, cells were detached and counted (passage 2). The time it took the cells to double in number was determined.

### Flow cytometry

After the second passage (cultivation period of app. 3 weeks), cells were detached by accutase (600 U/mL, Gibco/Life Technologies) from the tissue flasks, centrifuged at 460*g* for 5 min at RT, and resuspended in 50 μL PBS containing 3% (v/v) FCS. Aliquots of 1 × 10^6^ cells were incubated with antibodies against CD45 (V500, leukocyte common antigen, clone: HI30, Becton Dickinson), CD34 Class III (FITC, My10, clone: 581, Invitrogen, Thermo Fisher), CD73 (PerCP-eFlour-710, ecto-5-NT, SH4, clone: AD2, BD Bioscience), CD90 (Brilliant Violet 421, Thy-1, clone: 5E10, Bio Legend, Fell, Germany), and CD105 (PE-Cy7, Endoglin/TGF1-b3 receptor, clone: 43A3, Bio Legend) for 30 min on ice as described before [20, 26]. Isotype controls at the same concentration as the specific antibodies were used to determine nonspecific signals. FACS analysis was performed with a FACSCanto II flow cytometer (BD Bioscience) and Diva Software 6.0.

### Colony-forming unit (CFU) assay

2 × 10^6^ MNC of each group (BM, CB, VF, SR) were cultivated in a T25 tissue flask (cell density 4 × 10^5^ MNC/cm^2^). The medium was changed after 3 days. At day 7, cells were washed with PBS, fixed and incubated in 5% Giemsa solution (Merck, Darmstadt, Germany) for 5 min followed by rinsing with *aqua dest*. The colony-forming units were identified and counted using a stereomicroscope (Stemi 305, Zeiss, Jena, Germany). Colonies were defined as cell circular arrangement of more than 50 stained cells indicating that one viable cell gave rise to a colony through replication.

### Differentiation into the three lines

Mesenchymal multipotency was approved by applying typical in vitro stimulation protocols with the respective media followed by representative cytochemical staining as described before [4, 27]. In all groups, unstimulated cells served as control.

- Osteogenic differentiation: 1.8 × 10^4^ cells were cultivated in a six-well dish in an osteogenic medium. After 21 days, mineralization of the extracellular matrix was stained by Alizarin red.
- Chondrogenic differentiation: A cell pellet containing 3 × 10^4^ cells was made by centrifugation at 360*g* for 5 min and cultured in chondrogenic media in 96-well plates. After 21 days, the cell pellet was rinsed with PBS, overlayed with cooling-freezing media, and snap frozen in liquid nitrogen. Specimens were cut and stained with Alcian blue for glycosaminoglycans.
- Adipogenic differentiation: 1.8 × 10^4^ cells were cultivated in a six-well dish in adipogenic medium. After 21 days, adipocytes were detected by Oil Red O staining.

### Reverse transcription quantitative PCR (RT qPCR)

RNA was isolated from osteogenically stimulated cells after 7 and 21 days of culture with the RNeasy Mini Kit Plus (Qiagen, Hilden, Germany), which was applied according to the manufacturer’s protocol. Unstimulated cells served as controls. The concentration and purity of RNA was measured spectrophotometrically (NanoDrop™ Thermo Fisher). RNA was reversely transcribed to cDNA using a cDNA Synthesis Kit and Oligo (dT) primers according to the manufacturer’s protocol (Qiagen). *Quantitative PCR* (qPCR) was performed using SybrGreen, the DNA kit (Qiagen), and the iQ™ Cycler (Bio-Rad, München, Germany). All samples were analyzed as duplicates and had to show a clear melting curve including a characteristic peak. The target genes were normalized to the reference gene GAPDH using the der ΔΔ t method with ΔCt = Ct test gene − Ct reference gene (GAPDH) and ΔΔCt = ΔCt sample − ΔCt calibrator (unstimulated cells). The relative quantification (RQ) is the -fold change compared to the calibrator and was calculated as 2^-ΔΔCt^. A RQ of 10 means that this gene is 10 times more expressed in sample *x* than in the calibrator sample. We considered a RQ significant when there was a minimum of twofold change.

### Statistics

Statistical analysis was performed using Graph Pad Prism software V8 (GraphPad Prism Software, Inc. San Diego, CA). Continuous variables (patients’ age, sample weight, MNC, number) are presented as mean ± standard deviation and categorical variables (gender, comorbidities) as frequency and percentage. Ordinal parameters (CFU number) and continuous parameters (MNC and MSC number, generation time) are expressed as mean with the interquartile range (25th percentile-75th percentile). Analysis of normal distribution of each continuous variable was performed by the *Kolmogorov-Smirnov* test before further statistical testing. Accordingly, the *Kruskal-Wallis* test by ranks was used for the comparison of nonparametric values between the four study groups. Differences were considered significant at *p* < 0.05.

## Results

### Yield of mononuclear cells

**Fig. 2:**
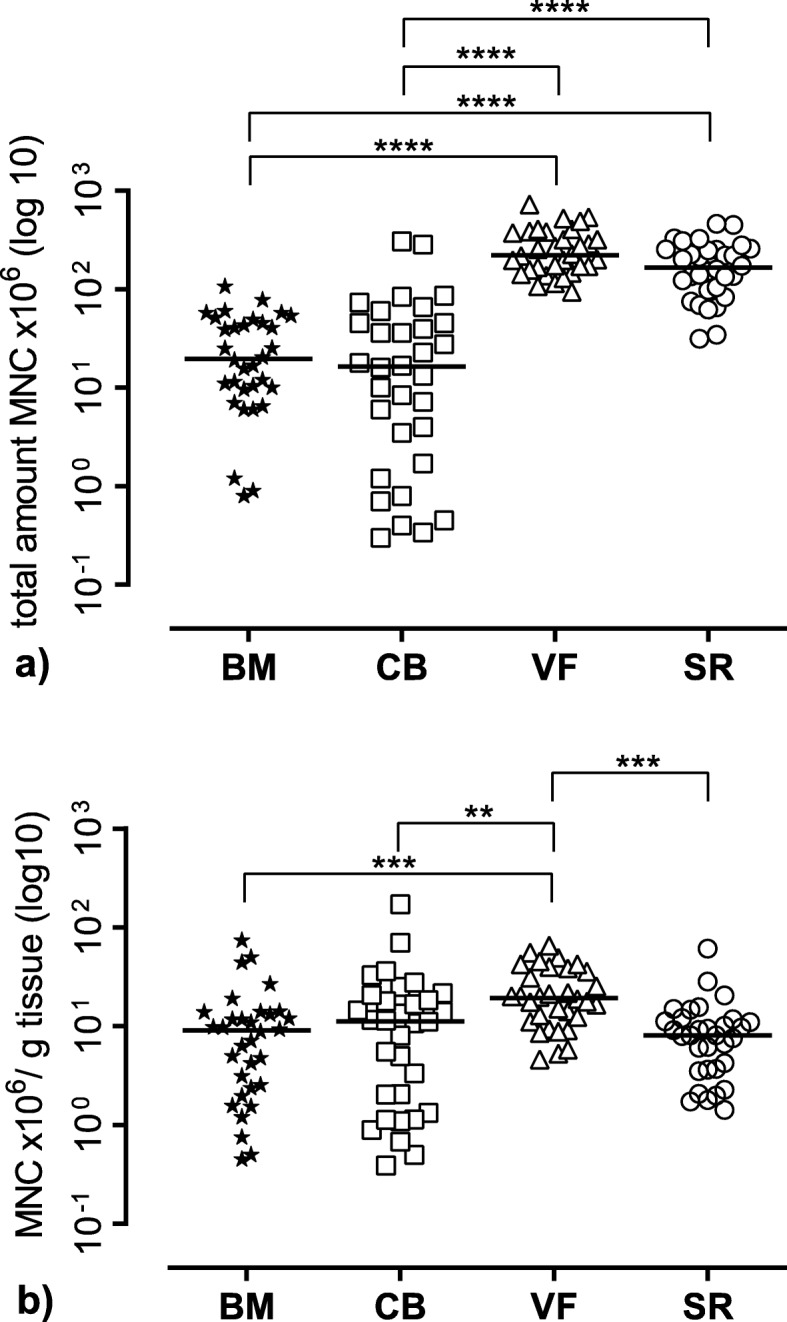
Mononuclear cell (MNC) yield of the different tissue sources. **a** Total number of harvested MNC of the different tissues. **b** The number of MNC per gram tissue sample. The figure shows the single values of *n* = 32 patients as symbols with the median as line. BM bone marrow, CB cancellous bone, VF vacuum filter, SF cell saver filtrate reservoir. Significant differences (Kruskal-Wallis test) are indicated with ****p < 0.0001, ***p < 0.001, and **p < 0.01

The average weight of the different tissue samples harvested in 32 patients was as follows: bone marrow aspirate 3.4 g ± 1.9 g [0.5–9.1], cancellous bone 3.3 g ± 6.6 g [0.3–28.5], surgical vacuum filter clot 14.3 ± 6.0 g [7.8–31.5], and cell saver reservoir 26.0 ± 14.3 g [9.3–69.1]. From all 32 patients, MNC could be isolated and cultured from all four groups (Table 2). Figure 2 a shows the total amount of harvested mononuclear cells (MNC) from the tissues, whereas b documents the MNC per gram tissue sample. Referring to the total amount of the isolated MNC, both groups of the surgical vacuum sucker (VF and SR) were superior to BM (*p* < 0.0001) and CB (*p* < 0.0001) (Fig. 2a). Moreover, the MNC number from VF and SR showed a smaller variance. Regarding the amount of MNC per gram tissue, the highest MNC concentration was found in VF, which was significantly higher than CB (*p* = 0.0096), BM (*p* = 0.0007), and SR (*p* = 0.0005) (Fig. 2b).

**Table 2:** Total number of MNC of the respective tissue source and number of MNC per gram tissue/fluid of *n* = 32 patients. Values are listed as mean ± SD and as median and interquartile range (IQR) calculated as the difference between 75th and 25th percentiles

	MNC × 10^6^ Mean ± SD Median (IQR)	MNC × 10^6^/g Mean ± SD Median (IQR)
BM	29.3 ± 25.6 19.7 (38.6)	12.4 ± 16.0 9.1 (11.3)
CB	41.3 ± 71.6 16.4 (43.5)	18.1 ± 31.6 11.3 (18.6)
VF	273.0 ± 149.9 222.5 (220)	23.8 ± 16.0 19.4 (26.5)
SR	188.4 ± 110.2 166.8 (154.9)	10.2 ± 11.1 8.1 (8.0)

### Proliferation potential

**Fig. 3:**
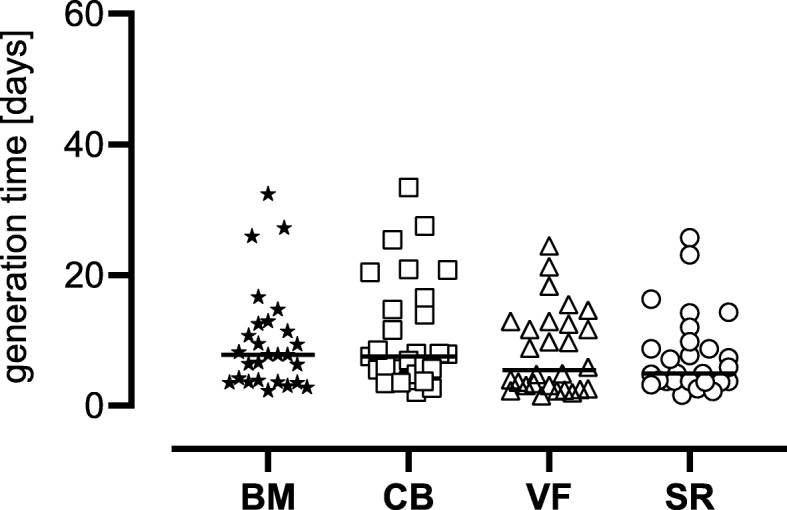
Generation time in days. The figure shows the single values (of *n* = 27 patients for BM and SR, of *n* = 28 patients for VF, and of *n* = 29 patients for CB) as symbols with the median as line. BM bone marrow, CB cancellous bone, VF vacuum filter, SF cell saver filtrate reservoir. There were no significant differences (Kruskal-Wallis test)

The proliferation potential determined as generation time was slightly lower for both groups of the surgical vacuum sucker (VF and SR) with 7.8 days for VF and 8.3 days for SR compared to 9.8 days for BM and 10.7 days for CB, but they were not statistically different (Table 3 and Fig. 3).

**Table 3:** Generation time of passage 1 and passage 2 of the adherent cells of the respective tissue source of at least *n* = 27 patients. Values are listed in days as mean ± SD and also as median, interquartile range (IQR) calculated as the difference between 75th and 25th percentiles and range

	Number	Mean ± SD	Median (IQR) [range]
BM	27	9.80 ± 7.81	7.8 (8.9) [2.3–32.4]
CB	29	10.74 ± 8.32	7.5 (10.45) [2.1–33.4]
VF	28	8.31 ± 6.43	5.4 (10.1) [1.5–24.5]
SR	27	7.81 ± 6.20	4.9 (6.1) [1.6–25.7]

### Differentiation potential: cell characterization by flow cytometry

**Fig. 4:**
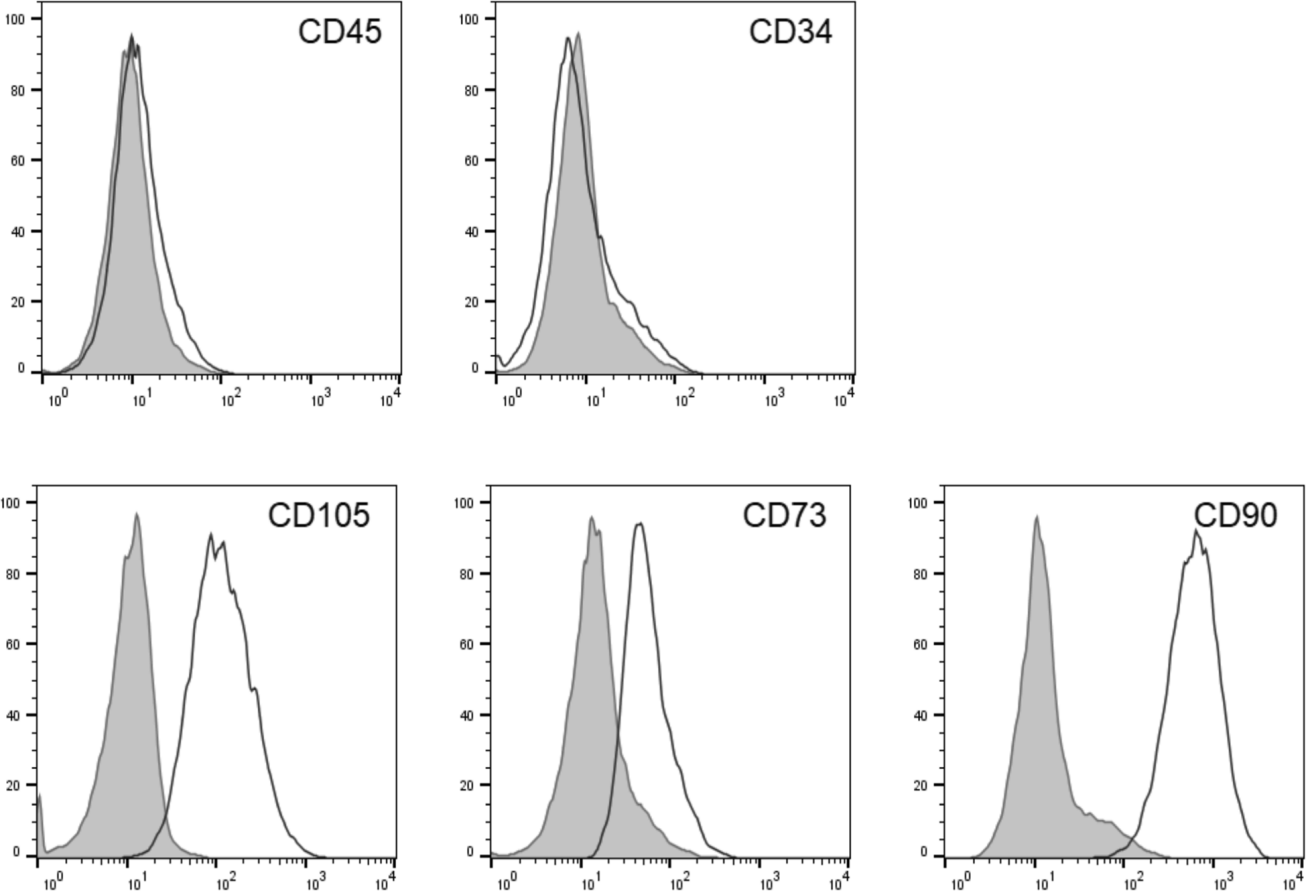
Representative flow cytometry analysis for all groups. Data are shown as a histogram overlay: isotype control (purple) and specific cell surface markers (blue line). Cells were labeled with antibodies against CD34, CD45, CD73, CD90, and CD105

The occurrence of MSC was controlled by typical expression markers via flow cytometry. Here, all samples showed a significant expression of the mesenchymal stromal cell markers (CD105^+^, CD73^+^, CD 90^+^) (Fig. 4). In contrast, cells expressed no hematopoietic markers (CD34^−^, CD45^−^). There were no differences of the expression type between the different groups.

### Differentiation potential: differentiation into osteoblasts, chondroblasts, and adipoblasts

**Fig. 5:**
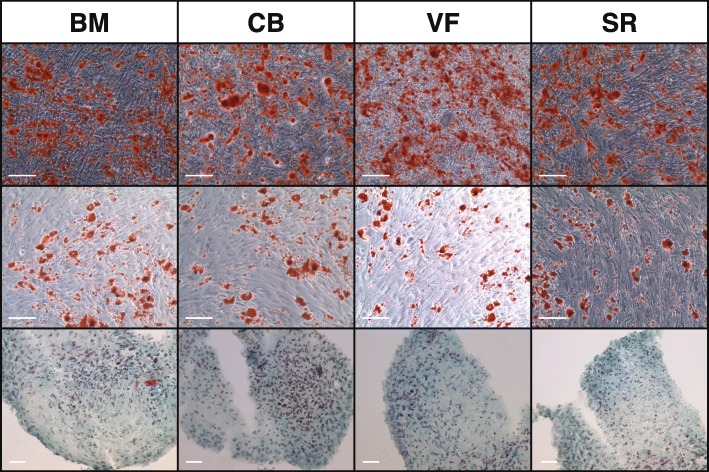
Characteristic cytochemical staining. Upper row: osteoblast differentiation: staining of calcium in the extracellular matrix components with Alizarin red. The calibration bar indicates 200 μm. Middle row: adipoblast differentiation: staining of intracellular neutral triglycerides with Oil Red. Calibration bar indicates 200 μm. Lower row: chondroblast differentiation: staining of glycosaminoglycans with Alcian blue. The calibration bar indicates 50 μm

No qualitative differences could be found in the lineage-specific differentiation between the different cell origins as could be shown by the characteristic cytochemical staining of calcium in the extracellular matrix components with Alizarin red (osteoblasts) or glycosaminoglycans with Alcian blue (chondroblasts) or intracellular neutral triglycerides with Oil Red (adipoblasts) (Fig. 5).

### Stemness character and MSC yield

**Fig. 6:**
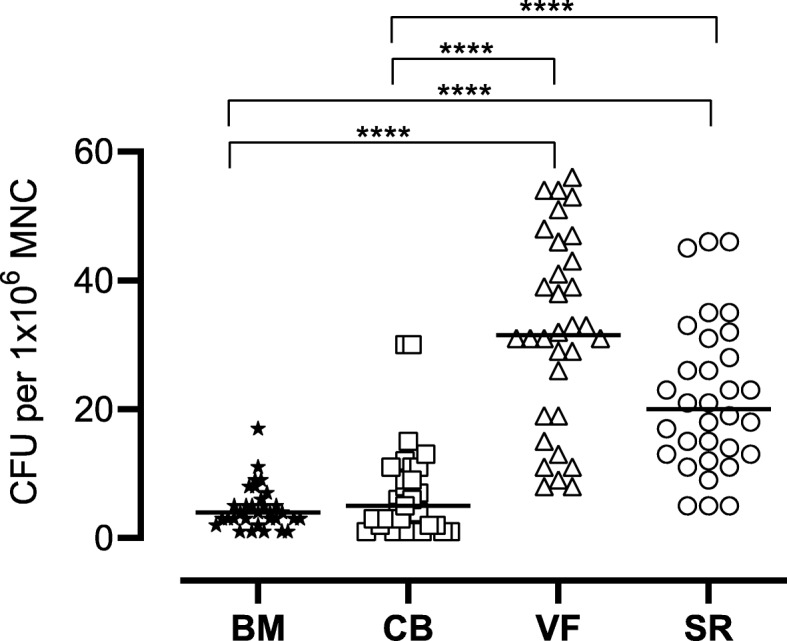
The number of colony-forming units (CFU) per 1 Mill mononuclear cells (MNC) after a cultivation period of 7 days. The figure shows the single values of *n* = 32 patients as symbols with the median as line. Significant differences (Kruskal-Wallis test) are indicated with ****p < 0.0001

A CFU assay was performed with mononuclear cells of each group (BM, CB, VF, and SR). We found the highest values in CFU for MNC harvested from the surgical vacuum sucker (VF and SR) compared to the bone marrow aspirate and cancellous bone. Especially, the cells cultivated from VF showed a higher number of CFU per 10^6^ MNC compared to SR (32.1 ± 15.0 vs. 21.7 ± 11.6) and a significantly higher one compared to CB (7.2 ± 7.3; *p* < 0.0001) and to BM (4.7 ± 3.4; *p* < 0.0001), respectively. Figure 6 documents the number of CFU after a cultivation period of 7 days.

**Fig. 7:**
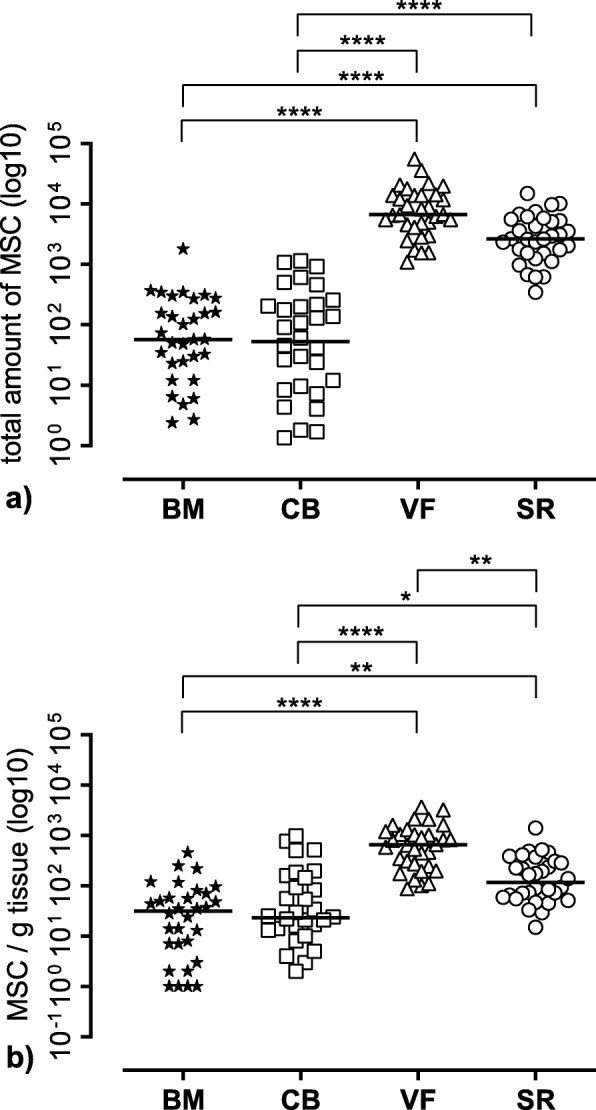
Mesenchymal stromal cell (MSC) yield of different tissue sources. **a** Total amount of MSC resulting from the initial mononuclear cell (MNC) number in the different tissue sources. **b** Amount of MSC per gram tissue weight resulting from the initial MNC number per gram tissue weight. The figure shows the single values of *n* = 32 patients as symbols with the median as line. BM bone marrow, CB cancellous bone, VF vacuum filter, SF cell saver filtrate reservoir. Significant differences (Kruskal-Wallis test) are indicated with ****p < 0.0001, ***p < 0.001, **p < 0.01, and *p < 0.05

From the CFU assay results, the number of MSC in 1 × 10^6^ MNC was determined and used for calculating the number of MSC in the different tissue samples (Table 4). The resulting total amount of MSC is shown in Fig. 7a, whereas Fig. 7b documents the number of MSC per gram tissue sample. With regard to the number of calculated MSC yield, we found the highest amount of MSC in the cell saver filtrate and the cell saver reservoir. Both VF and SR were superior to BM (*p* < 0.0001) and CB (*p* < 0.0001) (Fig. 7a). Moreover, MSC number from VF and SR showed a smaller variance. The highest tissue concentration of MSC was present in the vacuum filter, which was significantly higher compared to SR (*p* = 0.0034) vs. BM (*p* < 0.0001) and vs. CB (*p* < 0.0001) (Fig. 7b). Regarding the ratio of MSC per MNC, the values were 0.05‰ for BM, 0.07‰ for CB, 3.8‰ for VF, and 2.1‰ for SR.

**Table 4:** Total number of MSC resulting from MNC of the respective tissue source and number of MSC resulting from MNC per gram tissue/fluid of *n* = 32 patients. Values are listed as mean ± SD and as median and interquartile range (IQR) calculated as the difference between 75th and 25th percentiles

	MSC Mean ± SD Median (IQR)	MSC/g Mean ± SD Median (IQR)
BM	167.1 ± 324 57.4 (228.5)	59.2 ± 93.7 31.5 (62.7)
CB	203.9 ± 317.5 52.8 (208.5)	125.4 ± 235.5 23.0 (122.5)
VF	10,892 ± 11,152 6697 (9786)	899.2 ± 870.7 650.5 (898.7)
SR	3791 ± 3273 2643 (3985)	216.6 ± 264.3 116.0 (241.0)

### Comparison of osteoblastic marker expression

**Fig. 8:**
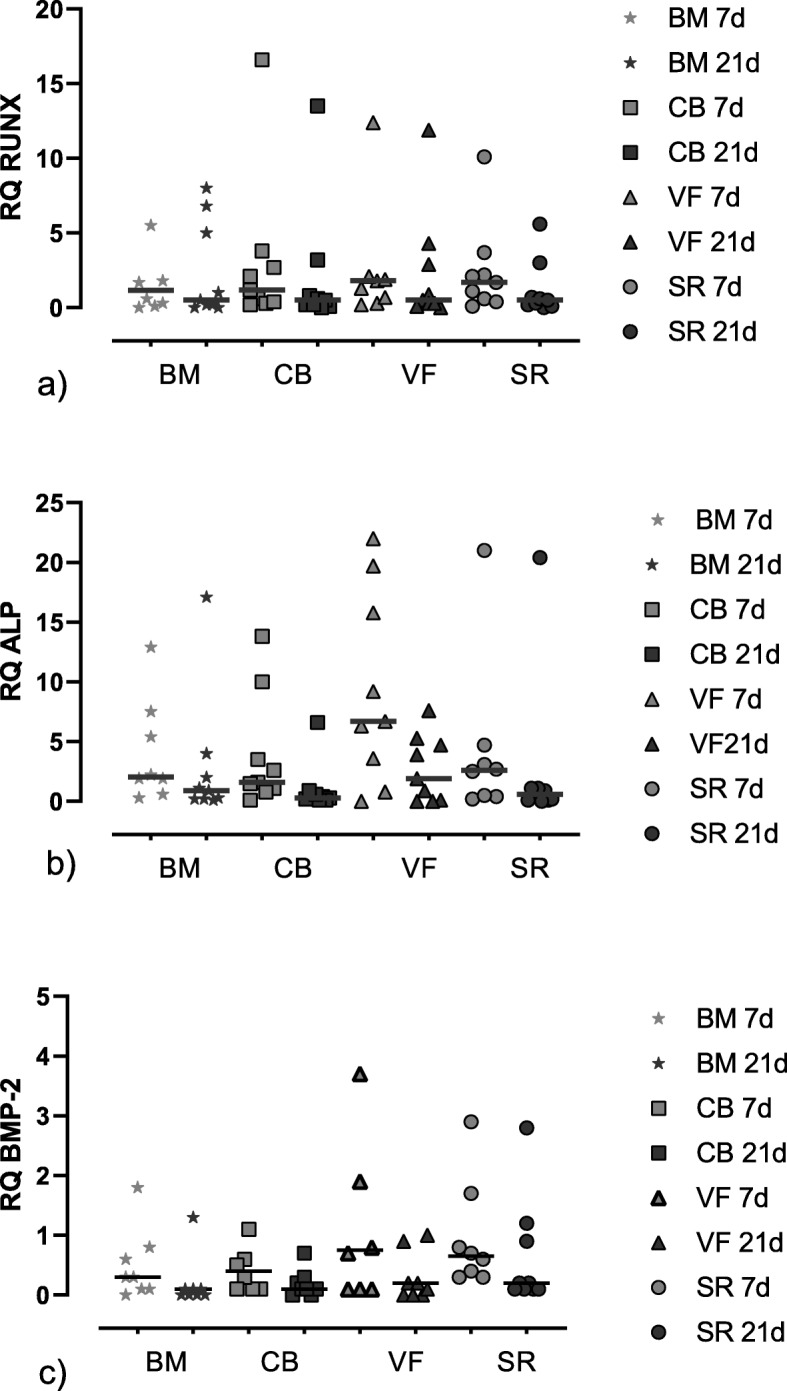
Gene expression of the osteoblastic markers. The target genes were normalized to the reference gene GAPDH using the der ΔCt method with ΔCt = Ct test gene − Ct reference gene (GAPDH) and ΔΔCt = ΔCt sample − ΔCt calibrator (unstimulated cells). The relative quantification (RQ) (=fold change compared to the calibrator) was calculated as 2^-ΔΔCt^. The figure shows the single values of *n* = 10 patients as symbols with the median as line. BM bone marrow, CB cancellous bone, VF vacuum filter, SF cell saver filtrate reservoir. **a** RQ of RUNX. **b** RQ of ALP. **c** RQ of BMP-2

The quantitative detection of the gene expression of osteoblastic markers RUNX-2, ALP, and BMP-2 was expressed as relative quantification (RQ) (=fold change compared to the calibrator = unstimulated cells) (Fig. 8). A RQ of 10 means that this gene is 10 times more expressed in sample *x* than in the calibrator sample. These markers were more expressed in the stimulated cells compared to the unstimulated cells at day 7 but not at day 21. There were no significant differences between the groups at day 7 nor at day 21.

## Discussion

This study clearly demonstrates that surgical vacuum filters are able to concentrate tissue with relevant amounts of MSCs. Compared to the other tissue sources (bone marrow aspirate and cancellous bone), more MNC and MSC per gram tissue were found in the cell saver system (vacuum suction handle, reservoir), but also the inter-individual variation in these samples was lower compared to the other tissues. The number of CFU per gram harvested tissue was also significantly higher in the surgical vacuum filter devices but showed a higher SD.

One limitation of this study is that we were not able to allocate the individual source of the MSC in the filter device. So, it remains unknown which tissue type is mainly responsible for the high number of the vacuum sucker-collected MSC. However, it is well known that adult stem cell niche is not limited to the bone marrow but varies in nature and location on the tissue type. Also, the technique of obtaining MSC by suction is not new. MSC derived by abdominal fat liposuction [26, 27] and MSC harvested by reamer-irrigator aspiration (RIA) [28] of human bone marrow from the iliac crest are typical examples for these techniques.

In our study, the source of the surgical vacuum sucker was a mixture of the bone marrow, bone, blood, fat, and soft tissue (skeletal muscle, connective tissue). In contrast to liposuction or RIA, it was obtained under a clinical in situ scenario (total hip implantation) without any additional invasive manipulation. Moreover, this mixture of different tissue component represents the composite which is initially adhering onto implant surface during total hip implantation [8].

But is a simple surgical vacuum suction handle really the best opportunity to collect or accumulate MSCs? In the present study, the yield in MNC and MSC of the different tissue sources was compared. For this, the fibrin clot adhered to the surface of the surgical vacuum filter was removed and had to be lysed. For this thrombolysis, an enzyme treatment with streptokinase was necessary. One limitation of this study is that this lysis was only performed in the cell saver system groups and not for the aspirate or cancellous bone group. We do not expect a negative influence of this treatment since we got the best yield in MSC for the cell saver system groups filter and reservoir. We controlled this issue and added the streptokinase incubation step into the isolation procedure of MSC from the cancellous bone (as described in list item 2 of the “Cell harvesting methods” section). We could not detect any difference in the number and viability of the MNC (*n* = 3). According to a previous study, systemic streptokinase administration had no significant effect on the number CD34/CXCR4+ stem cells [29]. In vitro studies revealed that the addition of 2000 IE/mL streptokinase prevented a decrease in the viability of cells in a mature (14 days) dissociated culture from the neocortex of rat pups induced by transfer of the cultures to medium lacking serum proteins [30]. When added to the culture of sensitive and sympathetic ganglia, streptokinase increased the proliferation of Schwann cells [31]. Therefore, it is unlikely that streptokinase treatment of surgical vacuum sucker groups (VF and SR) affected the cells in our system. However, even MSCs seem to be one key piece of the puzzle, it is not the only factor in tissue regeneration. Soluble factors such as cytokines and growth factors, nutrients, salts, and components of the extracellular matrix play also an important role in bone repair and tissue regeneration. It is conceivable that small pieces of local tissue combined with adsorbed proteins, growth factors, and other components might present a well-balanced and stronger regenerative tool than a cell therapeutic only. Although we found no correlation between patient-related factors (age, sex, comorbidities) and the evaluated in vitro parameters, we cannot exclude that individual factors might have an impact for the outcome.

In this context, it is unclear if streptokinase treatment might have an influence on the regenerative potential when applied not only to cells but to human tissue.

Regarding the gene expression described as RQ values, we detected the expression of osteoblastic markers also in the unstimulated controls after 21 days although no mineralization could be detected histologically. This striking fact that MSCs undergo in vitro mainly osteogenic differentiation through a well-defined pathway has been described before [32]. The group of Hernigou et al. analyzed MSC amplification (P1) in media without differentiation agents and the osteoblastic gene expression by quantitative RT-PCR [33]. They showed that the expression of ALP, bone sialoprotein, osteoprotegerin, and BMP-2 was spontaneously induced and significantly upregulated in cells cultured in platelet lysate compared to those in FCS at day 21. Interestingly, at the same time, no mineralization was detected without osteogenic stimuli [33]. This is in line with our data for the negative controls.

Looking at the yield of MSC from the bone marrow aspirate, Hernigou et al. evaluated the number and concentration of progenitor cells, which were transplanted for the treatment of nonunion [34]. After 4 months, callus volume obtained and the clinical healing rate were determined [34]. While their average yield was 612 ± 134 progenitor cells/mL, for bone union, about 55,000 progenitors were injected [34]. The authors could show a significant positive correlation between the volume of mineralized callus at 4 months and the number and concentration of fibroblast colony-forming units in the graft [34]. In the present study, the number of progenitor cells was 60 MSC/g for the bone marrow, 125 MSC/g for the cortical bone, and 217 MSC/g the filter reservoir. The vacuum filter provided about 900 MSC/g tissue, even more than that described for the bone marrow by Hernigou et al. [34].

Pettine et al. reported an average of 121 Mill total nucleated cells per milliliter bone marrow aspirate with 2713 CFU-F/mL (2.2‰) [35]. Patients receiving more than 2000 CFU-F/mL experienced a significant reduction in lumbar discogenic pain determined via disability index and visual analog scale [35]. Compared to these data, less mononucleated cells (10–23 Mill) were harvested per gram tissue but with a comparable ratio of MSC to MNC especially within the vacuum filter system (3.8‰ for VF and 2.1‰ for SR). This ratio seems to be higher when using bone marrow aspirate concentration systems: Scarpone et al. compared three different systems in 30 patients and calculated a MSC to MNC ratio of about 8.2‰ depending on donor age [36].

Moreover, it is evident that not only the number of cells applied to the patient might have an impact of the healing potency but the plating density itself should be considered to be a critical factor for the enrichment of “primitive” cells from heterogeneous sources. Especially, Sox 2 seems to orchestrate fine line between differentiation and proliferation [37, 38].

But are MSCs and its progenitors really useful for clinical application? One of the major challenges is a lack of standardization in MSC manufacturing for clinical application. A recent multicenter study showed that differences in manufacturing affect the characteristics and functions of human bone marrow stromal cells [39]. In that context, our approach meets the consensus recommendation of the AAOS/NIH for minimally manipulated autologous cell preparations (MMACP) [40].

The initial cellularity of the samples is an important factor [41]. In bone tissue engineering, BM-MSCs have often been isolated and expanded in vitro to obtain the desired cell number for seeding on scaffolds [42]. The cells were differentiated using appropriate chemical stimuli aimed at enhancing the mineralized ECM formation [43]. However, as soon as the cells are seeded in vitro, they are exposed to a completely unknown environment which exhibits a complex architecture as well as differentiating stimuli that are distinct to their native conditions [41]. Such a method may reveal to be effective in terms of final mineral matrix deposition, but it is still far from mimicking any physiological bone formation [44]. Researchers have started to investigate the key role exerted by the native ECM molecules and neighboring tissue population. They report that the microenvironment, also known as cell dynamic biomimetic osteogenic niche, consists of cell-secreted extracellular matrix (ECM) molecules. In this niche, a broad spectrum of cells exists, which cross talks, interacts, and affects the MSC fate in different ways [41].

Moreover, it is evident for clinical application that progenitor cells may have less immunogenic potency than fully differentiated cells. The risk for autoimmunity is lower if undifferentiated cells are used for cell therapy [45].

**Fig. 9:**
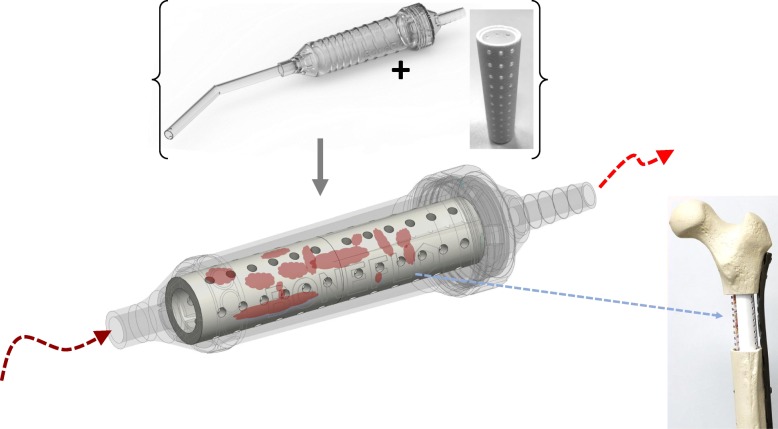
As an outlook for potential clinical application, an innovative surgical sucker handle was designed (EU/EFRE grant 1803su003). It includes a removable filter of beta-tricalcium-phosphate qualified for bone substitution and a biocompatible cylindric handle system with a connector and twist-off cap (BoneFlo®, TissueFlow GmbH, Germany)

Taking together the ethical and medico-legal issues as well as clinical aspects such as the patient’s safety, feasibility, and costs, we believe that an autologous, non-invasive generated tissue regenerate has a strong potential for clinical application in the next future. The reasons for this are primarily that it represents loco-typical cells and tissue components, it is located at the patient’s situs, and it is protected in a closed sterile system minimizing the infection risk. Transplanting different tissue components including characteristic cells, cytokines, and extracellular matrix components could create a healthy paracrine environment, providing additional support to promote functionality of transplanted cells. Especially, the combination of these tissue components with osteoconductive material such as synthetic bone graft might be an innovative and prospective technology. On the basis of the “tissue-flow” concept, we (MJ, MaH, AB) recently designed an innovative sucker handle made of PMMA including a removable TCP filter (BoneFlo®, granted by the EU). This tool considers recent medico-legal and as well as clinical aspects. It prevents further manipulation of the harvested tissue and provides a ready-to-use tool for the treatment of local bone defects (Fig. 9). However, clinical trials have to prove that this strategy is effective in bone regeneration.

Embarking on a strategy of minimal manipulation in autologous tissue engineering, it was shown in a randomized controlled clinical trial that the injection of autologous micro-fragmented adipose tissue is a safe and a valid therapeutic option improving healing rate in diabetic feet [46].

To date, it is not clear how regenerative medicine in orthopedics will develop in the future. As what happened in the past, uncharacterized cell products should not be declared and marketed as “stem cells” leading to a widespread clinical use of unproven biologic therapies [47, 48].

## Conclusion

We conclude that surgical vacuum filters are able to concentrate tissue with relevant amounts of MSCs. A new potent source of autologous MSC is identified. Further clinical studies have to elucidate the regenerative potential of these cells in an autologous setting.

## Data Availability

The datasets supporting the conclusions of this article are included within the article.

## Abbreviations

ALP: Alkaline phosphatase
BM: Bone marrow
BMP-2: Bone morphogenic protein 2
CB: Cancellous bone
CD: Cell surface antigens (CD34, CD45, CD73, CD90, CD105)
CFU: Colony-forming units
CXCR4: Chemokine receptor specific for stromal-derived-factor-1 (SDF-1)
ECM: Extracellular matrix
FCS: Fetal calf serum
GAPDH: Glycerol aldehyde dehydrogenase
MMACP: Minimally manipulated autologous cell preparations
MNC: Mononuclear cells
MSC: Mesenchymal stromal cells
PBS: Phosphate-buffered saline
PRP: Platelet-rich plasma
RIA: Reamer-irrigator aspiration
RQ: Relative quantification
RUNX2: Runt-related transcription factor 2
SF: Cell saver filtrate reservoir
VF: Vacuum filter

## References

[1] Yoo Jung U., Johnstone Brian (1998). The Role of Osteochondral Progenitor Cells in Fracture Repair. Clinical Orthopaedics and Related Research.

[2] Zhou Q, Yang C, Yang P (2017). The promotional effect of mesenchymal stem cell homing on bone tissue regeneration. Curr Stem Cell Res Ther.

[3] Garg P, Mazur MM, Buck AC, Wandtke ME, Liu J, Ebraheim NA (2017). Prospective review of mesenchymal stem cells differentiation into osteoblasts. Orthop Surg.

[4] Jäger M, Hernigou P, Zilkens C, Herten M, Fischer J, Krauspe R (2010). Cell therapy in bone-healing disorders. Orthopade..

[5] Matsumoto T, Kuroda R, Mifune Y, Kawamoto A, Shoji T, Miwa M (2008). Circulating endothelial/skeletal progenitor cells for bone regeneration and healing. Bone..

[6] Bielby R, Jones E, McGonagle D (2007). The role of mesenchymal stem cells in maintenance and repair of bone. Injury..

[7] Dimitriou R, Tsiridis E, Giannoudis PV (2005). Current concepts of molecular aspects of bone healing. Injury..

[8] Jäger Marcus, Jennissen Herbert P., Haversath Marcel, Busch André, Grupp Thomas, Sowislok Andrea, Herten Monika (2019). Intrasurgical Protein Layer on Titanium Arthroplasty Explants: From the Big Twelve to the Implant Proteome. PROTEOMICS – Clinical Applications.

[9] Egan KP, Duque G, Keenan MA, Pignolo RJ (2018). Circulating osteogentic precursor cells in non-hereditary heterotopic ossification. Bone..

[10] Haversath M, Catelas I, Li X, Tassemeier T, Jager M (2012). PGE(2) and BMP-2 in bone and cartilage metabolism: 2 intertwining pathways. Can J Physiol Pharmacol.

[11] Kan C, Chen L, Hu Y, Lu H, Li Y, Kessler JA (2017). Microenvironmental factors that regulate mesenchymal stem cells: lessons learned from the study of heterotopic ossification. Histol Histopathol.

[12] Kjaersgaard-Andersen P, Sletgard J, Gjerloff C, Lund F (1990). Heterotopic bone formation after noncemented total hip arthroplasty. Location of ectopic bone and the influence of postoperative antiinflammatory treatment. Clin Orthop Relat Res.

[13] Xu L, Liu Y, Sun Y, Wang B, Xiong Y, Lin W (2017). Tissue source determines the differentiation potentials of mesenchymal stem cells: a comparative study of human mesenchymal stem cells from bone marrow and adipose tissue. Stem Cell Res Ther.

[14] Buget MI, Dikici F, Edipoglu IS, Yildiz E, Valiyev N, Kucukay S (2016). Two-year experience with cell salvage in total hip arthroplasty. Braz J Anesthesiol.

[15] Eindhoven GB, Diercks RL, Richardson FJ, van Raaij JJ, Hagenaars JA, van Horn JR (2005). Adjusted transfusion triggers improve transfusion practice in orthopaedic surgery. Transfus Med.

[16] Hong KH, Pan JK, Xie H, Guo D, Yang WY, Su HT (2017). Review: autologous blood transfusion drainage compared with no drainage in total knee arthroplasty: a meta-analysis and systematic review. Pak J Pharm Sci.

[17] Pawaskar A, Salunke AA, Kekatpure A, Chen Y, Nambi GI, Tan J (2017). Do autologous blood transfusion systems reduce allogeneic blood transfusion in total knee arthroplasty?. Knee Surg Sports Traumatol Arthrosc.

[18] Rasouli MR, Maltenfort MG, Austin MS, Waters JH, Parvizi J, Erkocak OF (2016). Blood management after total joint arthroplasty in the United States: 19-year trend analysis. Transfusion..

[19] Horstmann WG, Swierstra MJ, Ohanis D, Rolink R, Kollen BJ, Verheyen CC (2014). Favourable results of a new intraoperative and postoperative filtered autologous blood re-transfusion system in total hip arthroplasty: a randomised controlled trial. Int Orthop.

[20] Cui R, Rekasi H, Hepner-Schefczyk M, Fessmann K, Petri RM, Bruderek K (2016). Human mesenchymal stromal/stem cells acquire immunostimulatory capacity upon cross-talk with natural killer cells and might improve the NK cell function of immunocompromised patients. Stem Cell Res Ther.

[21] Bourin P, Bunnell BA, Casteilla L, Dominici M, Katz AJ, March KL (2013). Stromal cells from the adipose tissue-derived stromal vascular fraction and culture expanded adipose tissue-derived stromal/stem cells: a joint statement of the International Federation for Adipose Therapeutics and Science (IFATS) and the International Society for Cellular Therapy (ISCT). Cytotherapy..

[22] Krampera M, Galipeau J, Shi Y, Tarte K, Sensebe L (2013). Therapy MSCCotISfC. Immunological characterization of multipotent mesenchymal stromal cells--the International Society for Cellular Therapy (ISCT) working proposal. Cytotherapy..

[23] Dominici M, Le Blanc K, Mueller I, Slaper-Cortenbach I, Marini F, Krause D (2006). Minimal criteria for defining multipotent mesenchymal stromal cells. The International Society for Cellular Therapy position statement. Cytotherapy..

[24] Horwitz EM, Le Blanc K, Dominici M, Mueller I, Slaper-Cortenbach I, Marini FC (2005). Clarification of the nomenclature for MSC: the International Society for Cellular Therapy position statement. Cytotherapy..

[25] Alvarez-Viejo M, Menendez-Menendez Y, Otero-Hernandez J (2015). CD271 as a marker to identify mesenchymal stem cells from diverse sources before culture. World J Stem Cells.

[26] Brandau S, Jakob M, Bruderek K, Bootz F, Giebel B, Radtke S (2014). Mesenchymal stem cells augment the anti-bacterial activity of neutrophil granulocytes. PLoS One.

[27] Herten M, Grassmann JP, Sager M, Benga L, Fischer JC, Jager M (2013). Bone marrow concentrate for autologous transplantation in minipigs. Characterization and osteogenic potential of mesenchymal stem cells. Vet Comp Orthop Traumatol.

[28] Kuehlfluck P, Moghaddam A, Helbig L, Child C, Wildemann B, Schmidmaier G (2015). RIA fractions contain mesenchymal stroma cells with high osteogenic potency. Injury..

[29] Abdallah KO, Saleh RM, Al-Shawarby LA, Amer HA, Mostafa S (2014). Detection of CD34/CXCR4+ stem cells in peripheral blood of patients following acute myocardial infarction. Egypt J Immunol.

[30] Nikandrov VN, Zhuk ON (2006). Effects of streptokinase on the development of rat cerebral cortical cells in vitro. Neurosci Behav Physiol.

[31] Nikandrov VN, Zhuk ON, Gronskaia RI, Polukoshko EF, Romanovskaia AA (2008). Effects of plasminogen and streptokinase on the vital functions of nervous tissue cells in culture. Biomed Khim.

[32] Vilamitjana-Amedee J, Bareille R, Rouais F, Caplan AI, Harmand MF (1993). Human bone marrow stromal cells express an osteoblastic phenotype in culture. In Vitro Cell Dev Biol Anim.

[33] Chevallier N, Anagnostou F, Zilber S, Bodivit G, Maurin S, Barrault A (2010). Osteoblastic differentiation of human mesenchymal stem cells with platelet lysate. Biomaterials..

[34] Hernigou P, Poignard A, Beaujean F, Rouard H (2005). Percutaneous autologous bone-marrow grafting for nonunions. Influence of the number and concentration of progenitor cells. J Bone Joint Surg Am.

[35] Pettine KA, Murphy MB, Suzuki RK, Sand TT (2015). Percutaneous injection of autologous bone marrow concentrate cells significantly reduces lumbar discogenic pain through 12 months. Stem Cells.

[36] Scarpone M, Kuebler D, Chambers A, De Filippo CM, Amatuzio M, Ichim TE (2019). Isolation of clinically relevant concentrations of bone marrow mesenchymal stem cells without centrifugation. J Transl Med.

[37] Yoon DS, Kim YH, Jung HS, Paik S, Lee JW (2011). Importance of Sox2 in maintenance of cell proliferation and multipotency of mesenchymal stem cells in low-density culture. Cell Prolif.

[38] Ding D, Xu H, Liang Q, Xu L, Zhao Y, Wang Y (2012). Over-expression of Sox2 in C3H10T1/2 cells inhibits osteoblast differentiation through Wnt and MAPK signalling pathways. Int Orthop.

[39] Liu S, de Castro LF, Jin P, Civini S, Ren J, Reems JA (2017). Manufacturing differences affect human bone marrow stromal cell characteristics and function: comparison of production methods and products from multiple centers. Sci Rep.

[40] Chu CR, Rodeo S, Bhutani N, Goodrich LR, Huard J, Irrgang J (2019). Optimizing clinical use of biologics in orthopaedic surgery: consensus recommendations from the 2018 AAOS/NIH U-13 conference. J Am Acad Orthop Surg.

[41] Danti S, Serino LP, D'Alessandro D, Moscato S, Danti S, Trombi L (2013). Growing bone tissue-engineered niches with graded osteogenicity: an in vitro method for biomimetic construct assembly. Tissue Eng Part C Methods.

[42] Jaiswal N, Haynesworth SE, Caplan AI, Bruder SP (1997). Osteogenic differentiation of purified, culture-expanded human mesenchymal stem cells in vitro. J Cell Biochem.

[43] Kasper FK, Liao J, Kretlow JD, Sikavitsas VI, Mikos AG (2008). Flow perfusion culture of mesenchymal stem cells for bone tissue engineering.

[44] Grayson WL, Martens TP, Eng GM, Radisic M, Vunjak-Novakovic G (2009). Biomimetic approach to tissue engineering. Semin Cell Dev Biol.

[45] Kode JA, Mukherjee S, Joglekar MV, Hardikar AA (2009). Mesenchymal stem cells: immunobiology and role in immunomodulation and tissue regeneration. Cytotherapy..

[46] Lonardi R, Leone N, Gennai S, Trevisi Borsari G, Covic T, Silingardi R (2019). Autologous micro-fragmented adipose tissue for the treatment of diabetic foot minor amputations: a randomized controlled single-center clinical trial (MiFrAADiF). Stem Cell Res Ther.

[47] Piuzzi NS, Ng M, Chughtai M, Khlopas A, Ng K, Mont MA (2018). The stem-cell market for the treatment of knee osteoarthritis: a patient perspective. J Knee Surg.

[48] Turner L, Knoepfler P (2016). Selling stem cells in the USA: assessing the direct-to-consumer industry. Cell Stem Cell.

